# Application of Continuous Intraoperative Neuromonitoring During VATS Lobectomy for Left Lung Cancer to Prevent Recurrent Laryngeal Nerve Injury

**DOI:** 10.1038/s41598-020-61500-6

**Published:** 2020-03-13

**Authors:** Young Jun Chai, Jung-Man Lee, Yong Won Seong, Hyeon Jong Moon

**Affiliations:** 1 Department of Surgery, Seoul Metropolitan Government Seoul National University Boramae Medical Center, 20 Boramae-ro 5-gil, Dongjak-gu, Seoul Korea; 2 Department of Anesthesiology and Pain Medicine, Seoul Metropolitan Government Seoul National University Boramae Medical Center, 20 Boramae-ro 5-gil, Dongjak-gu, Seoul Korea; 3 Department of Thoracic and Cardiovascular Surgery, Seoul Metropolitan Government Seoul National University Boramae Medical Center, 20 Boramae-ro 5-gil, Dongjak-gu, Seoul Korea

**Keywords:** Nervous system, Outcomes research

## Abstract

We applied continuous intraoperative neuromonitoring (CIONM) during video-assisted thoracoscopic surgery (VATS) lobectomy for left lung cancer and evaluated its safety and usefulness. An electrode was attached to a double-lumen tube, and placed at vocal cord level to detect the EMG signal evoked by vocal cord movement. Before 4 L lymph node dissection, an automatic periodic stimulation device was applied to the vagus nerve to stimulate vagus nerve continuously. Surgery was suspended if the amplitude decreased lower than the threshold and was resumed when the amplitude recovered. Ten patients (6 male, 4 female) were enrolled. CIONM was successfully performed in all patients without technical failure, and there was no hemodynamic instability. Amplitude decreased below the threshold in four patients. One patient did not recover amplitude and experienced transient vocal cord palsy. In the three other patients, the amplitude recovered above the threshold and no vocal cord palsy occurred. The six patients who did not exhibit amplitude decrease experienced no vocal cord palsy. Our results suggest that CIONM may be applied safely for VATS left lobectomy and may be used to predict postoperative vocal cord function. This approach may be helpful to prevent RLN injury during VATS left lobectomy.

## Introduction

Recurrent laryngeal nerve (RLN) injury is one of the most common complications associated with lung cancer surgery, occurring in 15 to 31% of surgeries^1,2^. The left RLN, which is located more caudal and dorsal than the right RLN, is injured more frequently during lung surgery than the right RLN^1^. The left RLN is often embedded in the connective tissue at the level of the aortic arch and is difficult to visualize during surgery. In particular, for patients requiring upper mediastinal lymph node dissection, the risk of RLN injury is increased because the RLN passes through the lymph node compartment^3^.

Preserving RLN function after lung resection is important because RLN palsy is associated with poor quality of life due to voice dysfunction^4^, postoperative pneumonia, and higher mortality^5^. Routine postoperative laryngeal assessment after lung cancer surgery is advocated for early detection of complications associated with RLN palsy^6^.

Intraoperative neuromonitoring (IONM) is a technique where the RLN or vagus nerve (VN) are stimulated using a stimulating probe, and RLN function is monitored by detecting electromyography at the vocal cords. This technique is useful for preserving the functional integrity of the VN and RLN and enabling clinicians to predict postoperative vocal cord function during surgery^7,8^. While intermittent IONM provides information about the integrity of the nerve at the moment the probe is applied, continuous IONM (CIONM) stimulates the VN constantly to provide continuous feedback about the RLN and VN function. Continuous IONM is superior to intermittent IONM because it alerts the surgeon of imminent nerve injury in real time, allowing the surgeon to immediately cease the injurious maneuver and prevent further nerve injury^9–11^. In this study, we investigated the feasibility and safety of applying CIONM during video-assisted thoracoscopic surgery (VATS) lobectomy for early-stage left lung cancer.

## Results

Ten patients (6 males and 4 females) were enrolled in the study, and CIONM was successfully performed on all patients. Patient demographics and clinicopathological characteristics are shown in Table 1. The mean patient age was 61.1 ± 8.2 years. Pathologic diagnoses were adenocarcinoma (n = 7), squamous cell carcinoma (n = 2), and lymphoepithelioma-like carcinoma (n = 1). The mean tumor size was 2.2 ± 0.8 cm.

**Table 1:** Patients characteristics.

Case	Sex	Age	BMI, kg/m^2^	Operation	Tumor size, cm	No. retrieved LNs at #4 L	Pathology
1	F	56	18.8	LLL	4.2	2	Lymphoepithelioma-like carcinoma
2	F	58	24.8	LUL	1.5	1	adenocarcinoma
3	M	60	21.3	LUL	2.9	6	adenocarcinoma
4	M	74	19.2	LUL	2.2	3	Squamous cell carcinoma
5	M	48	32.6	LUL	1.7	5	adenocarcinoma
6	M	54	21.5	LUL	2.5	4	adenocarcinoma
7	F	55	21.8	LUL	1.6	3	adenocarcinoma
8	F	64	27.6	LLL	1.5	7	adenocarcinoma
9	M	71	23.9	LLL	1.8	2	Squamous cell carcinoma
10	M	71	20.2	LLL	2.0	4	adenocarcinoma

LN; lymph node, LLL; left lower lobectomy, LUL; left upper lobectomy.

The CIONM results are shown in Table 2. Hemodynamic instability during CIONM did not occur in any cases. Thus, cardiovascular medication, such as inotropes, was not required. The median duration from vagus dissection to APS application was 6 minutes (range: 2–15) and the median baseline amplitude was 981 μV (range: 476–2700). The mean CIONM duration was 27 minutes (range: 21–52). There were adverse events (amplitude decrease) in four patients (case #1, #2, #3, #6), but vocal cord palsy occurred in the first patient only.

**Table 2:** Results of continuous intraoperative neuromonitoring.

	Values (n = 10)
Event of hemodynamic instability during CIOM, n	4
Intraoperative use of cardiovascular medications, n	0
Time for vagus dissection and APS application, min	6 (2, 15)
Baseline amplitude, μV	981 (476–2700)
Presence of adverse events	4
Amplitude before removal of APS, μV	955 (254, 1705)
Total CIONM time, min	27 (21, 52)
Postoperative vocal cord palsy	1

The values are presented as number or median (range).

CIONM; continuous intraoperative neuromonitoring, APS; automated periodic stimulation.

**Figure 1:**
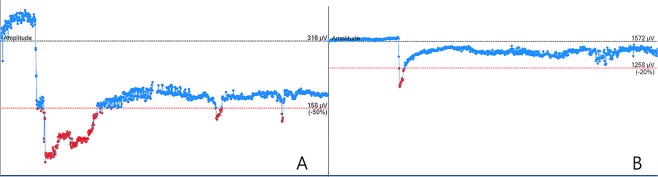
(**A)** Threshold for amplitude decrease is set at 50% of the baseline amplitude, and abrupt amplitude decrease was observed. (**B)** Threshold for amplitude decrease is set at 20% of the baseline amplitude, and an amplitude decrease of about 30% is observed but immediately recovers to more than 80% from baseline when surgery is suspended.

For the patient who developed vocal cord palsy, initial baseline amplitude was 2700 μV and amplitude abruptly decreased to 120 μV during 4L lymph node dissection. Surgery was suspended for 10 minutes, and the baseline amplitude was re-set to 316 μV. After resuming surgery, amplitude abruptly decreased a second time, and the final amplitude was 254 μV before APS removal (Fig. 1A). Postoperative laryngoscopic examination revealed left vocal cord palsy, which fully resolved in five months. The alarm threshold was then set at 20% decrease of amplitude for subsequent procedures. Three more patients (case #2, #3, #6) experienced adverse events. In the three cases, amplitude decreased about 30%, but recovered immediately when surgery was suspended, and vocal cord function remained unaffected (Fig. 1B).

## Discussion

Compared to open thoracotomy, VATS lobectomy is associated with less pain, less morbidity, shorter hospital stay, and early recovery, without compromising oncological outcomes^12–15^. Therefore, since the early 2000’s, VATS lobectomy has been a standard procedure for the treatment of early-stage non-small cell lung cancer (NSCLC)^16,17^. To the best of our knowledge, we are the first to report the application of CIONM during VATS lobectomy for left lung cancer, and demonstrate that CIONM can be safely applied and helpful for avoiding RLN injury.

Of the 10 patients in this study, the first patient experienced transient vocal cord palsy. That patient’s final amplitude was 254 μV which is less than 10% of their initial baseline amplitude. In this case, vocal cord palsy was predicted intraoperatively, because the RLN is likely to be injured if the amplitude does not recover more than 50% of baseline^18^. Because RLN injury occurred despite using CIONM, we made two modifications to prevent RLN injury in the future. First, we changed the threshold setting for amplitude decrease from 50% to 20% so that we could detect smaller amplitude decreases and suspend surgery as necessary. Second, before starting 4L lymph node dissection, we clearly visualized whole RLN from its origin at the VN using intermittent IONM. Using this method, we were able to apply energy-based devices at least 5 mm distant from the RLN to avoid RLN thermal injury. We believe that procedural modifications using CIONM for VATS may have resulted in decreased RLN injury and preserved function.

As suspected in the first case of this study, the most common injury mechanism of the RLN during thoracic surgery is stretching injury which occurs when the RLN or the tissue adjacent to the RLN is stretched^19^. During stretching injury, EMG amplitude shows a typical gradual decreasing pattern, which is reversible. If an amplitude decrease is detected during CIONM, the stretching forces applied around the RLN can be released to minimize nerve injury. The EMG reading can fully recover if stretching is relieved before complete loss of signal^20^. This is because the endoneurium as well as the epineurium and perineurium remain intact when the RLN is injured by stretching^21^. Therefore, it is important to detect small amplitude decreases to preserve RLN function.

Studies applying IONM for RLN and VN during open lung resection reported promising results^22,23^. Although in those studies intermittent IONM was useful for identifying RLN, it has limited clinical value because nerve integrity between stimulations cannot be assessed, and RLN injury cannot be prevented before it occurs^24,25^. In addition, to stimulate the nerve using stimulating probe during VATS is more time-consuming than during open surgery because the surgeon must halt the procedure to change instruments. Using CIONM in VATS is more efficient and helpful for preserving RLN than intermittent IONM.

The most common reason for IONM failure is malposition of the endotracheal tube^7^, Correctly placing the electrodes on the vocal cords is important for detecting a strong EMG signal. When intubating using an endotracheal tube during thyroid surgery, the anesthesiologist attaches the electrodes to the endotracheal tube and, using direct visualization, adjusts the depth of the tube so that the electrodes are placed at the vocal cords. A double-lumen endobronchial tube should be used for one-lung ventilation during VATS lobectomy. The bronchial tip of the tube should be fixed at the carina. Prior to intubation, it is difficult to know which part of the tube should be placed at the vocal cords. One study used preoperative CT scans to measure the distance between the true vocal cords and the main bronchus^22^. In the current study, after the first intubation, a fiberoptic bronchoscope was used to visualize and mark which part of the tube was placed at the arytenoid cartilage. The endobronchial tube was withdrawn and an adhesive electrode was attached

Continuous IONM in VATS for lung cancer may facilitate more complete lymph node dissection. Some surgeons advocate routine dissection of the N2 lymph nodes^26^, because the incidence of N2 lymph node metastasis ranges from 5–11.5%, even in clinically N0 lung cancer patients^27^. Compared to open thoracotomy, VATS has the disadvantage that complete N2 lymph node dissection can be complicated, owing to concerns about RLN injury. In fact, N1 and N2 lymph node metastases in clinically N0 lung cancer patients are more frequently detected after open thoracotomy than VATS^28^. In particular, dissection of station 4 L is challenging because of increased risk for RLN injury^29^, and the lymph nodes are often left behind. Although there is still a disagreement as to whether to perform station 4 L dissection in early-stage lung cancer, station 4 L dissection may be performed more safely if CIONM is used. In this sense, CIONM may enhance oncological outcomes as well as the surgical safety of VATS and open thoracotomy. Additional procedures and time are required for CIONM. Compared to the routine VATS procedure without nerve monitoring, this study required the following additional five steps: (1) checking the marking (letter or numeral) on the tube at the level of the arytenoid cartilage using a fiberoptic bronchoscope, (2) extubating, (3) cleansing the tube, (4) attaching electrode on the tube so that it would be placed 1.5 cm below the arytenoid, and (5) reintubation. This five-step process took around 3 to 5 minutes. In addition to the extended anesthesia time, this approach has an increased risk of airway injury due to the second intubation. To avoid the risk associated with the second intubation, the use of a single lumen tube with a surface electrode and a bronchial blocker is an alternative which requires only single intubation, but carries additional risks (such as cuff herniation). In addition, CIONM requires the circumferential VN dissection, which took average 6 minutes in this study. While the procedure itself has been shown to be safe^11^, VN damage can occur during nerve dissection or APS placement^30^. Surgeons should take great care while manipulating the VN and the APS. Another possible CIONM-related complication is hemodynamic instability due to continuous vagal stimulation^31^. Therefore, we excluded patients with uncontrolled hypertension, a history of coronary artery disease, or cerebrovascular disease. During the study, there was no airway injury, VN injury, or hemodynamic instability.

The CIONM technique also requires additional equipment and materials, including a monitoring system and stimulating probe as well as adhesive electrode and APS, which vary in cost according to the insurance system and country where the procedure is performed. Moreover, additional training of the anesthesiology team and surgeons is required. Considering the incidence and clinical importance of vocal cord palsy, the cost-effectiveness of CIONM should be evaluated in the future.

Our study has limitations. First, surgery was performed by an experienced surgeon in patients with clinically N0 early-stage NSCLC. Therefore, the results of this study cannot be generalized to low volume surgeons or advanced stage NSCLC patients. Second, this study was not a randomized controlled study, and the number of participants was too small to demonstrate that CIONM during VATS lobectomy for left lung cancer can reduce the incidence of RLN injury. Randomized controlled studies comparing VATS with or without CIONM with larger patient populations are warranted.

In summary, CIONM could be applied safely to VATS lobectomy for left lung cancer. Our results offer a better understanding of the injury mechanisms of RLN, and how to help preserve the RLN during VATS lobectomy.

## Patients and Methods

### Patients

This prospective trial using CIONM during VATS left lobectomy on lung cancer patients was approved by the Institutional Review Board of Seoul Metropolitan Government Seoul National University Boramae Medical Center (IRB permit number: 2017-43-083). All methods were performed in accordance with the relevant guidelines and regulations. From May 2018 to March 2019, patients diagnosed with early-stage NSCLC by preoperative fine needle aspiration and CT scan, requiring VATS left lobectomy were recruited for this study. Due to the potential for hemodynamic instability, we excluded patients with, uncontrolled hypertension, a history of coronary artery disease, or cerebrovascular disease. Patients with vocal cord paralysis at preoperative laryngoscopic examination were also excluded. Patients were counseled about the use of CIONM during surgery and informed consent to participate in trail was obtained prior to surgery. Surgery was performed by a single experienced thoracic surgeon (H.J.M) who performed about 300 cases of VATS for lung cancer prior to starting this study.

### Preoperative preparation

Preoperative CT scan, PET scan, and endobronchial ultrasound were routinely performed for the evaluation of tumor and lymph node status. Preoperative indirect laryngoscopic examination was routinely performed to evaluate vocal cord function. Patients were admitted to the ward one day prior to the surgery. Prophylactic antibiotics (cefotetan 1 g) were administered intravenously 30 min prior to incision.

### Anesthesia

All anesthetic procedures were performed or supervised by a single anesthesiologist (J.L). Patients were brought into the operating room without premedication and positioned on the operating table in a supine position with the head resting on a foam pillow. Pulse oximetry, electrocardiography, and non-invasive arterial blood pressure were monitored. Anesthesia was induced with glycopyrrolate (0.2 mg), lidocaine (30 mg), propofol (1.5 mg/kg), and continuous infusion of remifentanil with target-controlled infusion system. After confirming loss of consciousness, the patient’s lungs were ventilated by manual bagging with oxygen and sevoflurane. Then rocuronium (0.8 mg/kg) was administered for muscle relaxation prior to intubation. A 20-gauge catheter was inserted into the radial artery for continuous arterial blood pressure monitoring.

**Figure 2:**
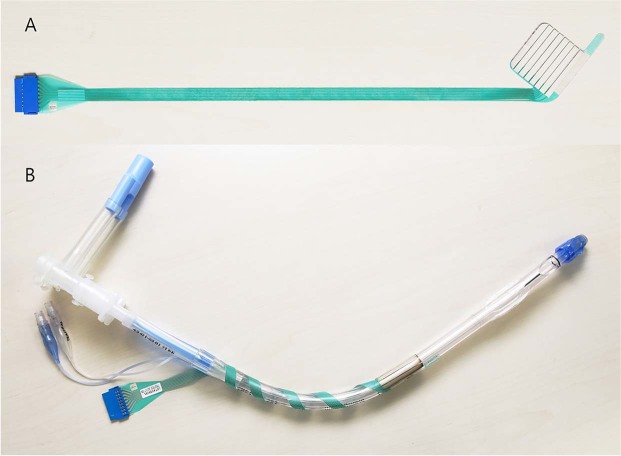
(**A**) Four-channel tube adhesive electrode before attachment. (**B**) Four-channel tube adhesive electrode after attachment on double-lumen tube.

**Figure 3:**
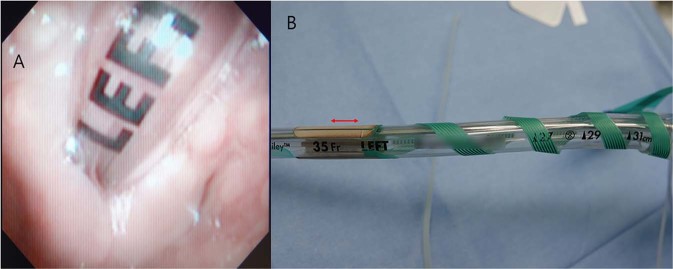
(**A**) Confirming which part of the double-lumen tube is placed at the level of the arytenoid cartilage using fiberoptic bronchoscope. (**B**) The center of the adhesive electrode is positioned 1.5 cm below the confirmed point (just below letter ‘L’) so that the center of the electrode is placed at the vocal cord.

For all patients, tracheal intubation for one-lung ventilation was performed with a left-sided double-lumen endobronchial tube (Mallinckrodt endobronchial tube; Covidien, Mansfield, MA). A four-channel tube adhesive electrode (Inomed, Emmendingen, Germany) was used to monitor electromyography (EMG) at the vocal cords (Fig. 2). The diameter of the double-lumen endobronchial tube and the size of the adhesive electrode were determined according to the inner diameter of the left main bronchus shown on preoperative CT scan. Intubation was performed twice in order to precisely position the electrode at the vocal cord level. The first intubation was performed using a 4.1 mm fiberoptic bronchoscope (Olympus LE-P; Olympus Optical Co. Tokyo, Japan) for visualization. The depth of the endobronchial tube was adjusted so that the bronchial tip of the tube was located at the carina. The tube was fixed at the mouth commissure when proper ventilation was confirmed. The anesthesiologist checked which part of the endobronchial tube was located at the level of the arytenoid cartilage using the fiberoptic bronchoscope (Fig. 3A), and extubated the patient. After cleansing the endobronchial tube with sterile gauze, an adhesive electrode was attached to the tube so that the center of the electrode would be placed 1.5 cm below the arytenoid cartilage (Fig. 3B), because the vocal cords are located approximately 1.2 to 1.5 cm below the arytenoid cartilage^32^. The anesthesiologist performed a second intubation to the depth determined at the first intubation. The final location of the bronchial tip of the endobronchial tube and the electrode were confirmed using the fiberoptic bronchoscope.

After inserting a central venous catheter, the patient was placed in the right lateral decubitus position. Rocuronium (10 mg) was administered hourly for muscle relaxation until the surgeon requested its discontinuation at least 30 minutes prior to performing CIONM. Glycopyrrolate (0.4 mg) and pyridostigmine (15 mg) were administered at the outset of CIONM to reverse muscle relaxation. Anesthesia was maintained with sevoflurane and continuous infusion of remifentanil. Tidal volume was initially set at 8 ml/kg and adjusted to about 5 ml/kg following one-lung ventilation. Respiration rate was set to obtain an arterial partial pressure of carbon dioxide of about 40 mmHg on arterial blood gas analysis.

### Equipment setting

All set up and monitoring was performed in compliance with standards outlined in the International Neural Monitoring Study Group guidelines^7^. We used the NIM Response 3.0 system (Medtronic, Jacksonville, FL, USA), 230 mm ball tip monopolar stimulating probe (Medtronic, Jacksonville, FL, USA) for intermittent stimulation, and automated periodic stimulation (APS) electrodes (3 mm; Medtronic, Jacksonville, FL, USA) for continuous stimulation. For intermittent stimulation, the event threshold was set at 100 mV, and the stimulus current at 1 mA, with a frequency of 4 Hz. For CIONM, the stimulus current was set at 1 mA, with a frequency of 1 Hz. The VN and RLN were considered to be successfully stimulated when the EMG amplitude was over 500 μV, during stimulation.

A decrease in amplitude or increase in latency indicates imminent RLN risk of injury and thus is considered to be an adverse event. In the first patient, the threshold for adverse event was set according to the manufacturer recommendations at 50% decrease of amplitude from baseline or 10% increase of latency from baseline, and the visual and acoustic alarm was triggered when an adverse event occurred. From the second patient onwards, we set the threshold at 20% decrease of amplitude from baseline or 10% increase of latency, to enable us to detect smaller amplitude decreases.

### Operative and nerve monitoring procedures

VATS lobectomy was performed while the patient was in the full left lateral decubitus position, using a utility incision and two 12 mm ports, in a similar manner as described in the literature^16^. A 3–5 cm utility incision was made at the fourth, fifth or sixth intercostal space along the anterior axillary line. A thoracoscopic port was made in the eighth intercostal space at the midaxillary or mid-posterior axillary line. The vessels and bronchi of the target lobe were individually dissected and divided with staplers. The resected lung was placed into an impermeable bag and removed through the utility incision. Systematic lymph node dissection of stations 4 L, 5, 6, 7, 9 L, 10 L, 11 L, and 12 L was then performed.

Using the stimulating probe, the location of the VN and the depth of neuromuscular blockade were evaluated before stations 4 L and 5 L lymph node dissection. If the amplitude was more than 500 μV, we began CIONM procedures. If the amplitude was less than 500 μV, lymph node dissection (which is not associated with risk of RLN injury) was commenced and the vagus nerve was subsequently stimulated regularly until the amplitude exceeded 500 μV. When satisfactory EMG amplitude was not achieved despite reversal of neuromuscular blockade, the tube location was adjusted by inserting or withdrawing the tube in 0.5 cm increments, while monitoring baseline amplitude.

**Figure 4:**
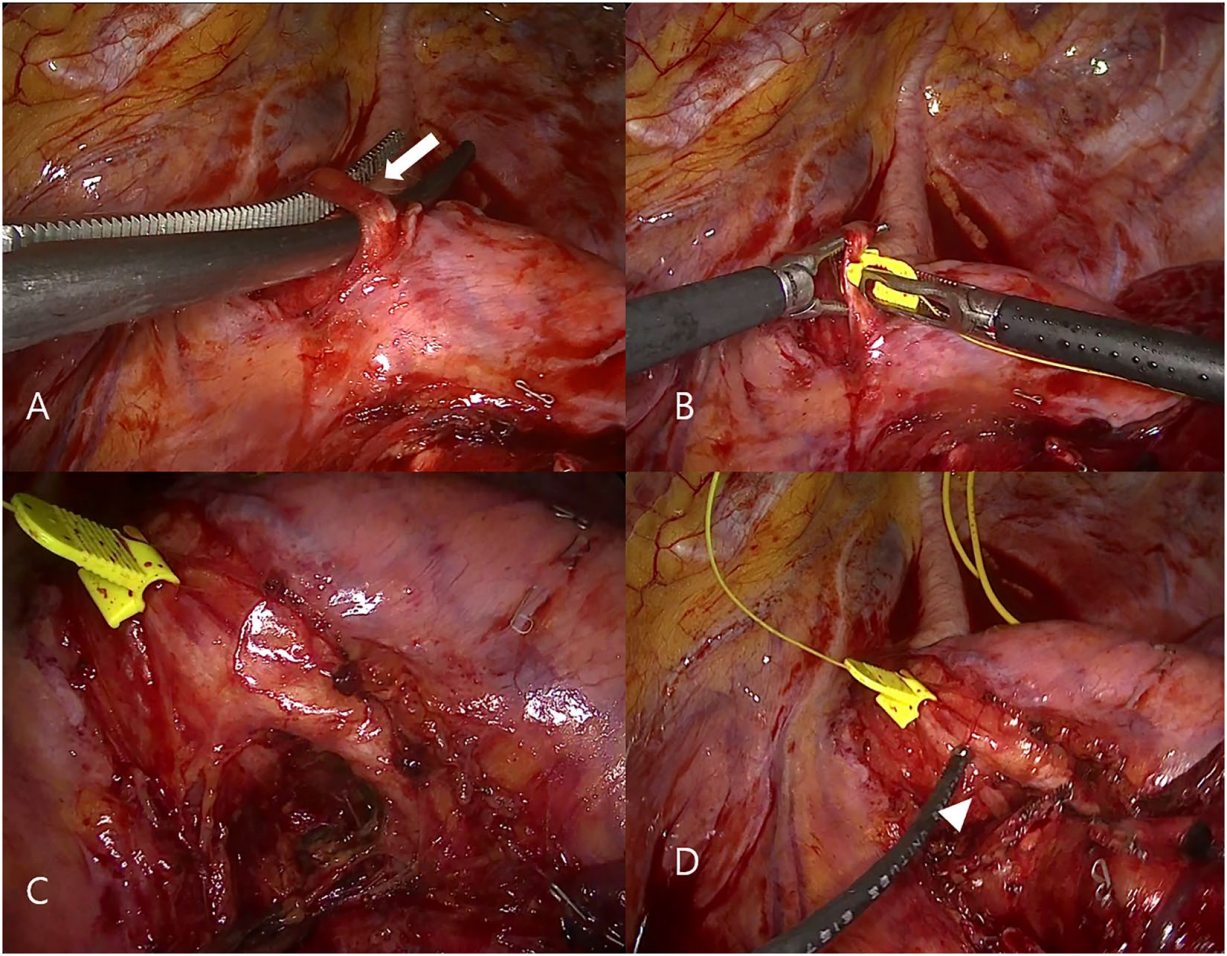
(**A**) Left vagus nerve (white arrow) was dissected circumferentially for a 2 cm segment. (**B**) Automatic periodic stimulation probe was applied. (**C**) Vagus and recurrent laryngeal nerve after 4 L lymph node resection. (**D**) Stimulating recurrent laryngeal nerve (arrow head) with stimulation probe to check functional integrity of the nerve.

For the CIONM procedures, the VN was dissected circumferentially in a 2 cm segment, and automated periodic stimulation was applied to the VN (Fig. 4). The baseline amplitude was set before 4 L lymph node dissection was performed. The stimulating probe was used to confirm the location of the RLN. When the alarm was triggered, surgery was suspended until the amplitude recovered above the threshold. Automated periodic stimulation was discontinued after 4 L lymph node dissection was complete.

### Checklist

During and after surgery, we recorded events of hemodynamic instability during CIONM (defined as systolic blood pressure <80 or >180 mmHg, diastolic blood pressure <40 mmHg, heart rate <40 or >120 bpm), time taken for vagus dissection and APS application, baseline amplitude, number of adverse events, and amplitude before removal of APS (Table 3).

**Table 3:** Check list for continuous intraoperative neuromonitoring.

1. Age/Sex	
2. Heigth and body weight	— — — — — cm — — — — — kg
3. Date of surgery	— — — — - — — - — —
4. Event of hemodynamic instability (SBP > 180 mmHg, SBP < 80 or DBP < 40 mmHg, HR > 120 or <40)	◻ no ◻ yes Please specify — — — — — — — — — — — — — —
5. Intraoperative use of cardiovascular medicaions	◻ no ◻ yes Please specify type, dose, injection time
6. Difficulties in intubation and extubation	◻ no ◻ yes please specify — — — — — — — — — — — — — —
7. Muscle relaxant (type, dose, injection time)	1)_______________________, _______mg/kg, ______:_______ 2)_______________________, _______mg/kg, ______:_______ 3)_______________________, _______mg/kg, ______:_______ 4)_______________________, _______mg/kg, ______:_______
8. Maintanance anesthesic agents	
9. Extent of lymph node dissection	
10. Time for vagus dissection and APS application	— — — — min
11. Baseline amplitude	— — — — mV
12. Adverse events (number, degree)	
13. Amplitude before removal of APS	
14. Total CIONM time	— — — — min
15. Reason for CIONM failure (if failed)	
16. Possible mechanism of RLN or VN injury (if present)	

SBP; systolic blood pressure, DBP; diastolic blood pressure, HR; heart rates, CIONM; continuous intraoperative neuromonitoring, APS; automated periodic stimulation, RLN; recurrent laryngeal nerve, VN; vagus nerve.

### Postoperative management and follow-up

Water intake was permitted eight hours postoperatively. A soft bland diet was initiated on postoperative day (POD) 1 and advanced to a regular diet on POD 2. The chest tube was removed when the volume of drainage was less than 200 ml per day, and the patient was discharged one day after tube removal. One week after discharge, patients returned to the outpatient clinic for stitch removal and indirect laryngoscopic examination by otolaryngologist. For patients with vocal cord palsy, vocal cord function was regularly monitored by laryngoscopic examination until full recovery was achieved.

## Data Availability

Authors agree to comply with the publication’s requirements for sharing materials.
